# Supplementary figures and images for: The mammalian Ire1 inhibitor, 4µ8C, exhibits broad anti-Aspergillus activity in vitro and in a treatment model of fungal keratitis
Source: Front Cell Infect Microbiol. 2024 Nov 12;14:1477463. doi: 10.3389/fcimb.2024.1477463 (PMC11588707; doi:10.3389/fcimb.2024.1477463)

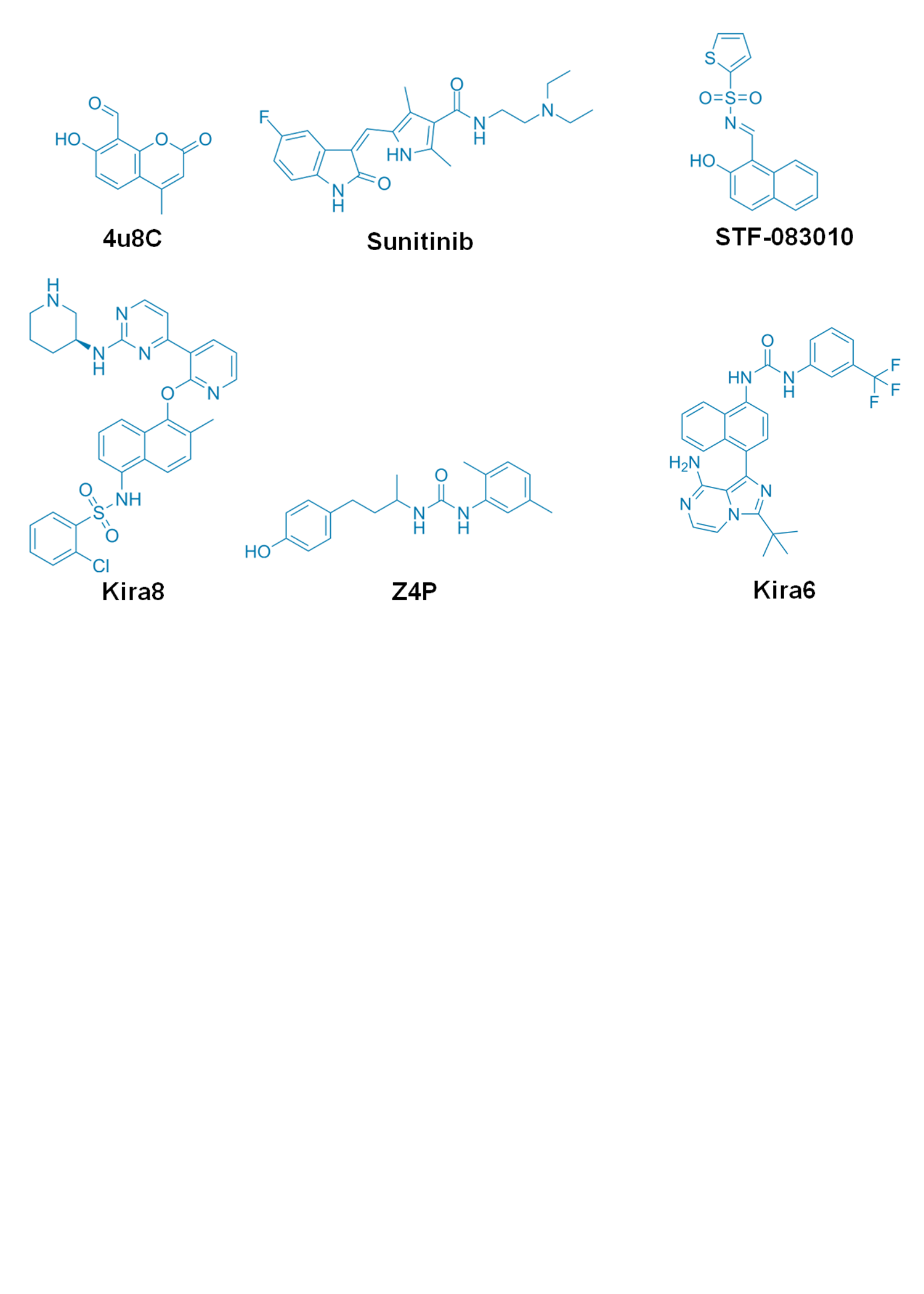

Supplement: Supplementary Figure 1 — Chemical structure of the drugs screened in this study. [file Image1.tif]

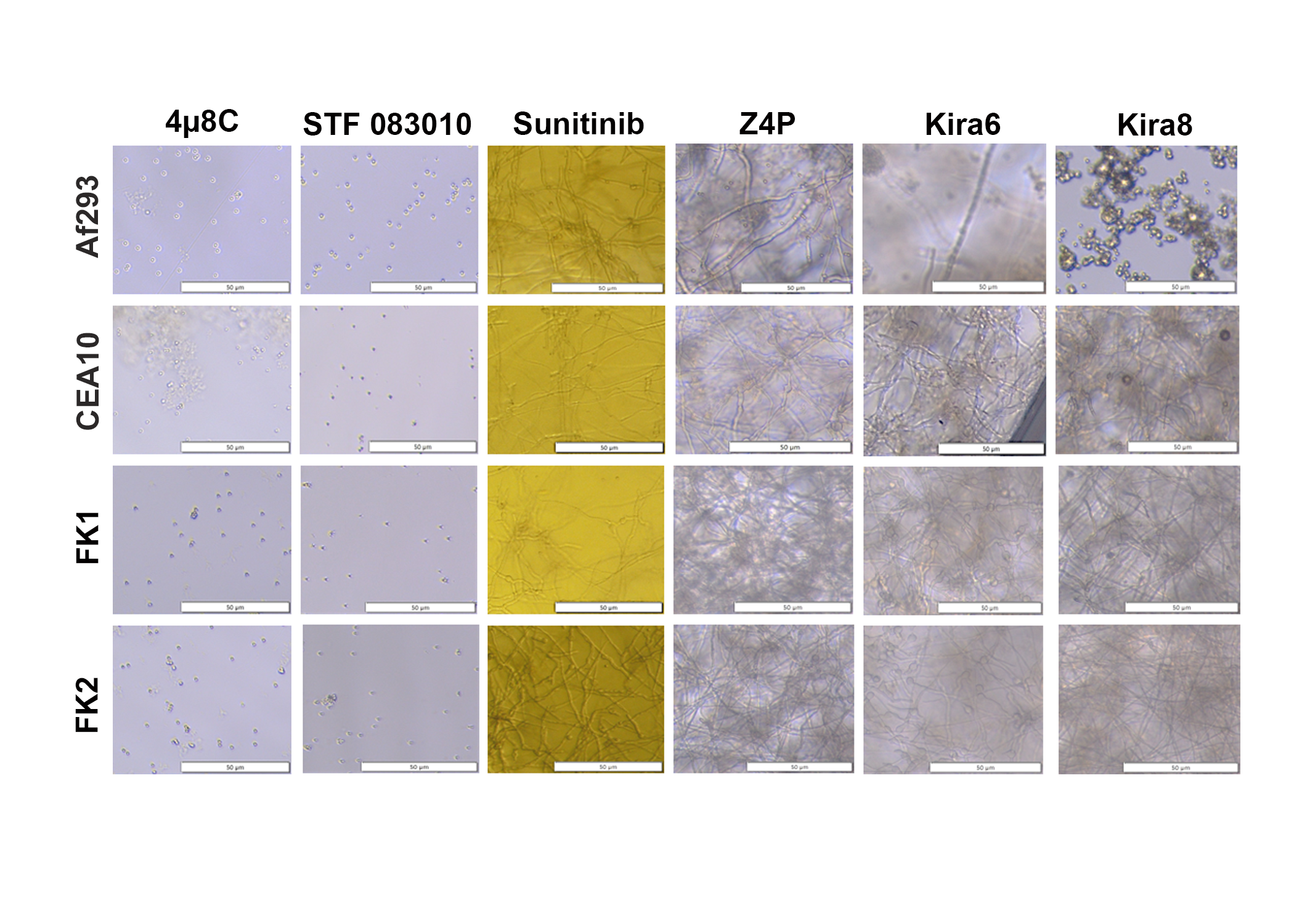

Supplement: Supplementary Figure 2 — The Ire1 kinase domain inhibitors do not display antifungal activity in vitro. Conidia of the A. fumigatus isolates were inoculated into GMM broth containing the 480 µM of the indicated drug and incubated for 72 h at 35°C. Images represent a consistent result observed across three independent experiments. [file Image2.tif]

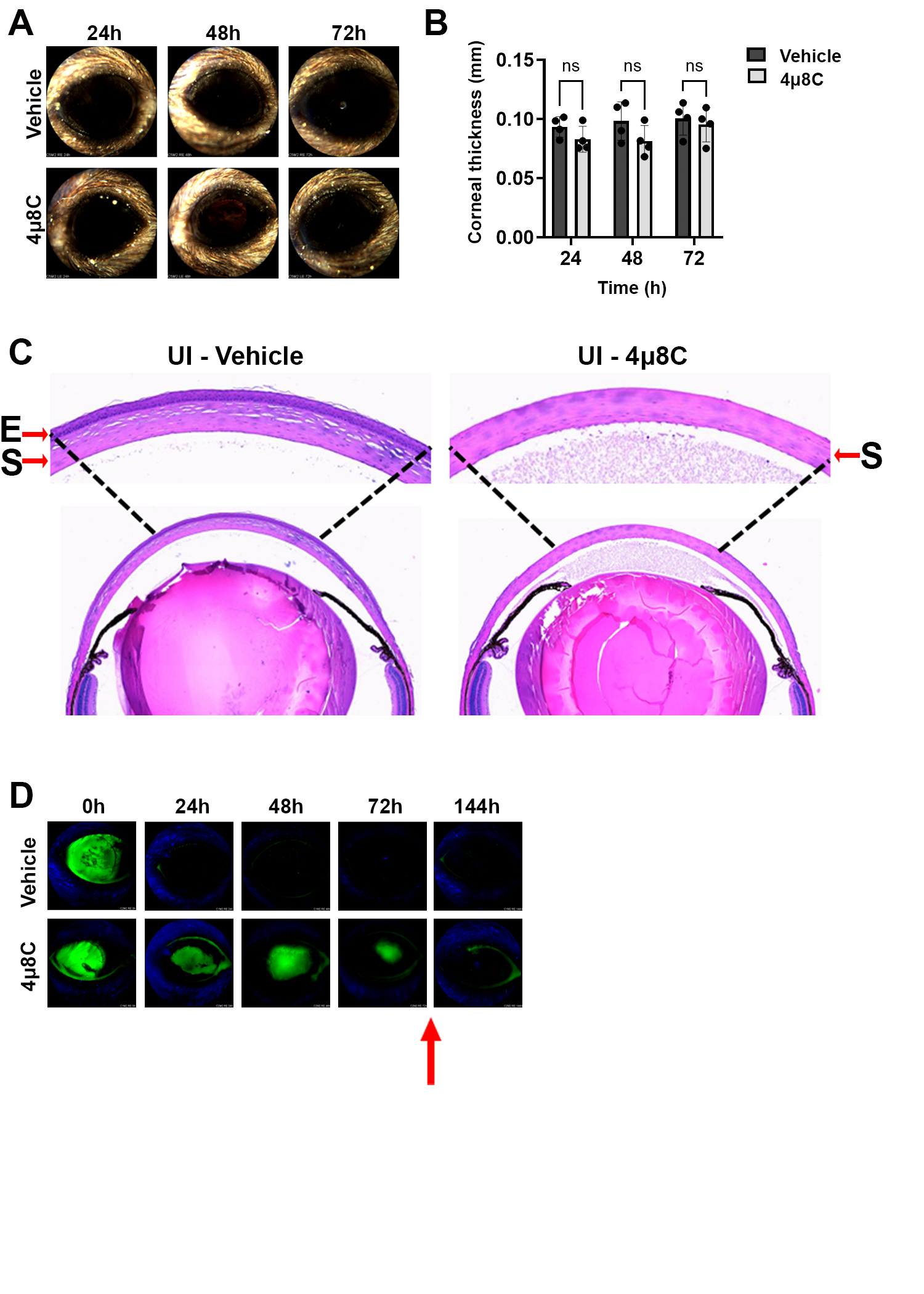

Supplement: Supplementary Figure 4 — 0.5 mM 4µ8C treatment does not impact corneal clarity but does transiently inhibit re-epithelialization. Sham-inoculated (UI) corneas were treated with topical drops of 0.5 mM 4µ8C or vehicle (DMSO) three times per day (4 h apart) starting on day after ulceration. (A) Representative external images taken each day post-ulceration. (B) Corneal thickness was measured daily based on OCT images. Groups were compared by Two-way ANOVA; (C) Representative histological (H&E) sections taken at 72 h post-ulceration; 400X magnification. The arrows are highlighting the epithelial ‘E’ and the stromal ‘S’ layers. (D) In a separate experiment, ulcerated eyes were treated as described above and on each day the penetration of fluorescein was imaged by a fluorescent slit-lamp (Micron IV). The red arrow indicates that the treatment was stopped, and the eyes were monitored for an additional three days. [file Image4.tif]

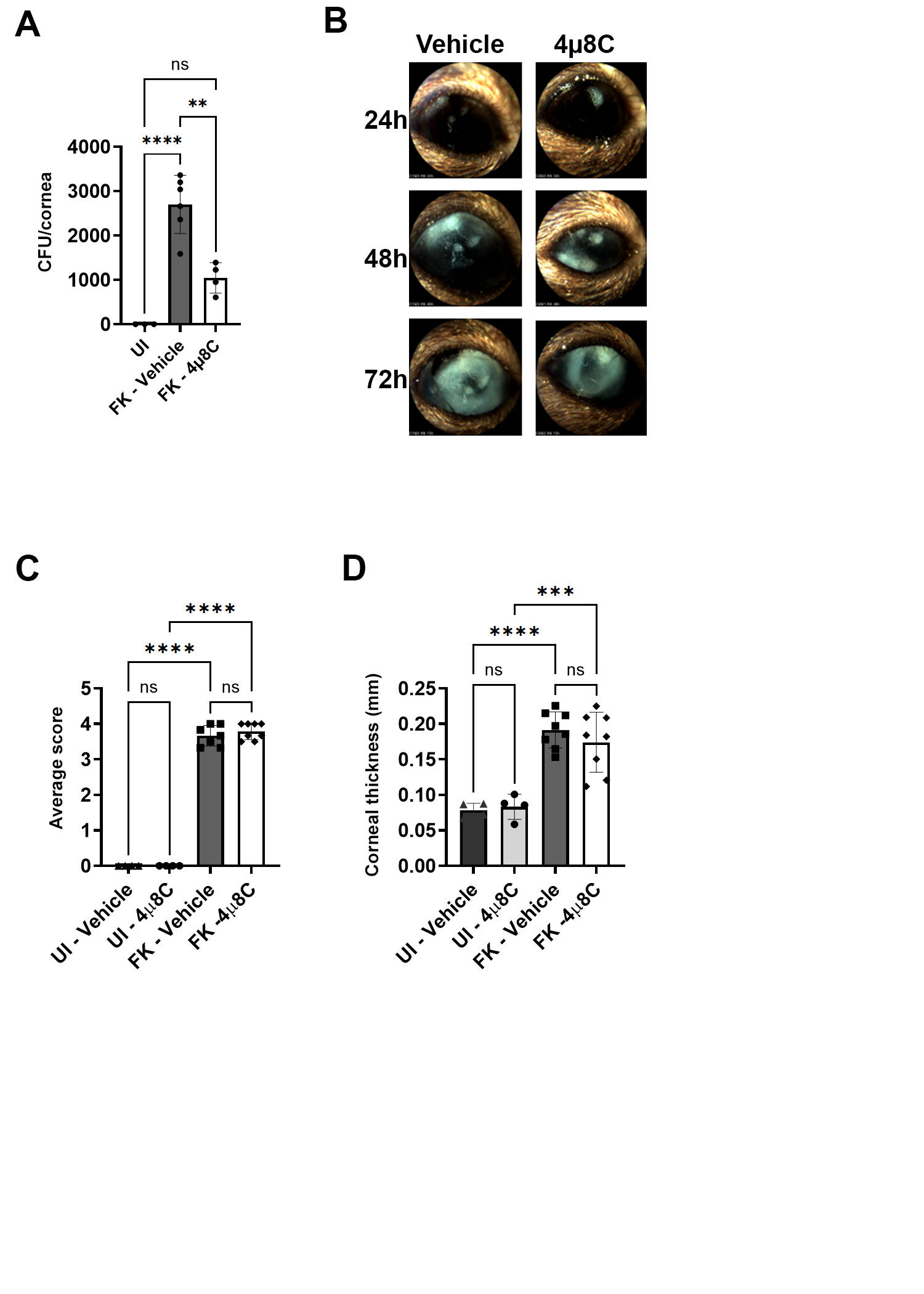

Supplement: Supplementary Figure 5 — Treatment with 0.5 mM 4µ8C reduces fungal burden but has no significant impact on clinical disease severity. Af293-inoculated (FK) corneas were treated with topical drops of 0.5 mM 4µ8C or vehicle (DMSO) 3X per day starting on day after ulceration and as described in the methods. The data reflect results from a single experiment (n=4 per UI group; n=8 per FK group). (A) Fungal burden at 72 h p.i. Groups were compared by Ordinary one-way ANOVA p-value **** <0.0001, ** 0.0012 (n=3, UI; n=6, FK-vehicle; n=4, FK-4µ8C); (B) Representative micron images over the course of the infection. (C) Average clinical scores at 72 h p.i. Groups were compared by Ordinary one-way ANOVA, p-value **** <0.0001; (C) Corneal thickness measurements at 72 h p.i. Groups were compared by Ordinary one-way ANOVA p-value **** <0.0001, *** 0.0003. [file Image5.tif]
